# Sclerosis of the clavicle––A challenging diagnosis^☆^

**DOI:** 10.1016/j.radcr.2022.04.012

**Published:** 2022-05-06

**Authors:** Ty A. Davis, Jacklyn Garcia, Thelma Rocio Jimenez Mosquea, Stephanie D. Zarate, Andrew A. Renshaw, Ana C. Belzarena

**Affiliations:** a Larkin Community Hospital, 7031 Southwest 62nd Avenue South Miami, FL, 33143, USA; b Herbert Wertheim College of Medicine, Florida International University, 11200 SW 8th St, Miami, FL, 33199, USA; c Universidad Iberoamericana, Av. Francia 129, Santo Domingo 10203, Dominican Republic; d Orthopaedic Oncology Department, Miami Cancer Institute, 8900 N Kendall Dr., Miami, FL, 33176, USA; e Department of Pathology, Baptist Hospital and Miami Cancer Institute, Miami, FL, 33176, USA

**Keywords:** Condensing osteitis, Clavicle, Bone sclerosis, Clavicle sclerosis

## Abstract

Condensing osteitis of the clavicle is a rare benign disease described as an increase in bone density at the medial end of the clavicle. Its clinical and radiographic presentation can frequently be equivocal and tissue sampling is necessary for diagnostic confirmation. Here we present the case of a 29-year-old female with condensing osteitis of the right medical clavicle, who remained undiagnosed for many years despite obtaining imaging studies and undergoing an initial biopsy. This disease presents oftentimes a challenging diagnosis due to its imaging features overlapping with many benign and malignant bone lesions. A qualified multidisciplinary team with expertise in rare bone conditions becomes oftentimes essential to arrive at an accurate diagnosis.

## Introduction

Condensing osteitis of the clavicle is a rare benign disease first described in 1974 as an increase in bone density at the medial end of the clavicle [1]. This disease presents in women of childbearing age, although male and pediatric patients have been described [2,3]. The clinical symptoms include edema and medial clavicle pain that worsens with abduction of the arm [1]. Osteonecrosis in the affected area, confirmed by biopsy, may suggests this uncommon diagnosis [4]. Some studies have identified a relationship between mechanical stress and degenerative changes of the sternoclavicular joint while others consider this a disease of infectious origin or even a true benign bone tumor; unfortunately, the ultimate etiology remains still unknown [1,[5], [6], [7].

Based on the presentation of symptoms, treatment indications can differ. In cases of asymptomatic or mild pain conservative management has been suggested [8,9]. However, in certain cases, there may be recommendation for anti-inflammatory drugs, antibiotics and local corticosteroid injections, depending on the particular case and manifestation [8]. Additional data advised a nonradical excision of the involved bone area in refractory scenarios [6,10]. Diagnosis via radiographic imaging studies exhibits sclerosis and expansion at the medial end of the clavicle, a highly unspecific finding sometimes misdiagnosed as a bone malignancy [1,6]. More advanced imaging studies such as CT scans or MRI demonstrate expansion of the medial clavicle, increased bone density in the area and edema [10]. The rarity of this disease, with scarcity of cases described in the literature, along its unspecific symptoms and imaging findings, with a myriad of differential diagnoses to be considered, can make its identification challenging for the treating physician. Here we described the case of a patient with worsening pain of the medial right clavicle that remained undiagnosed for years.

## Case report

**Fig. 1:**
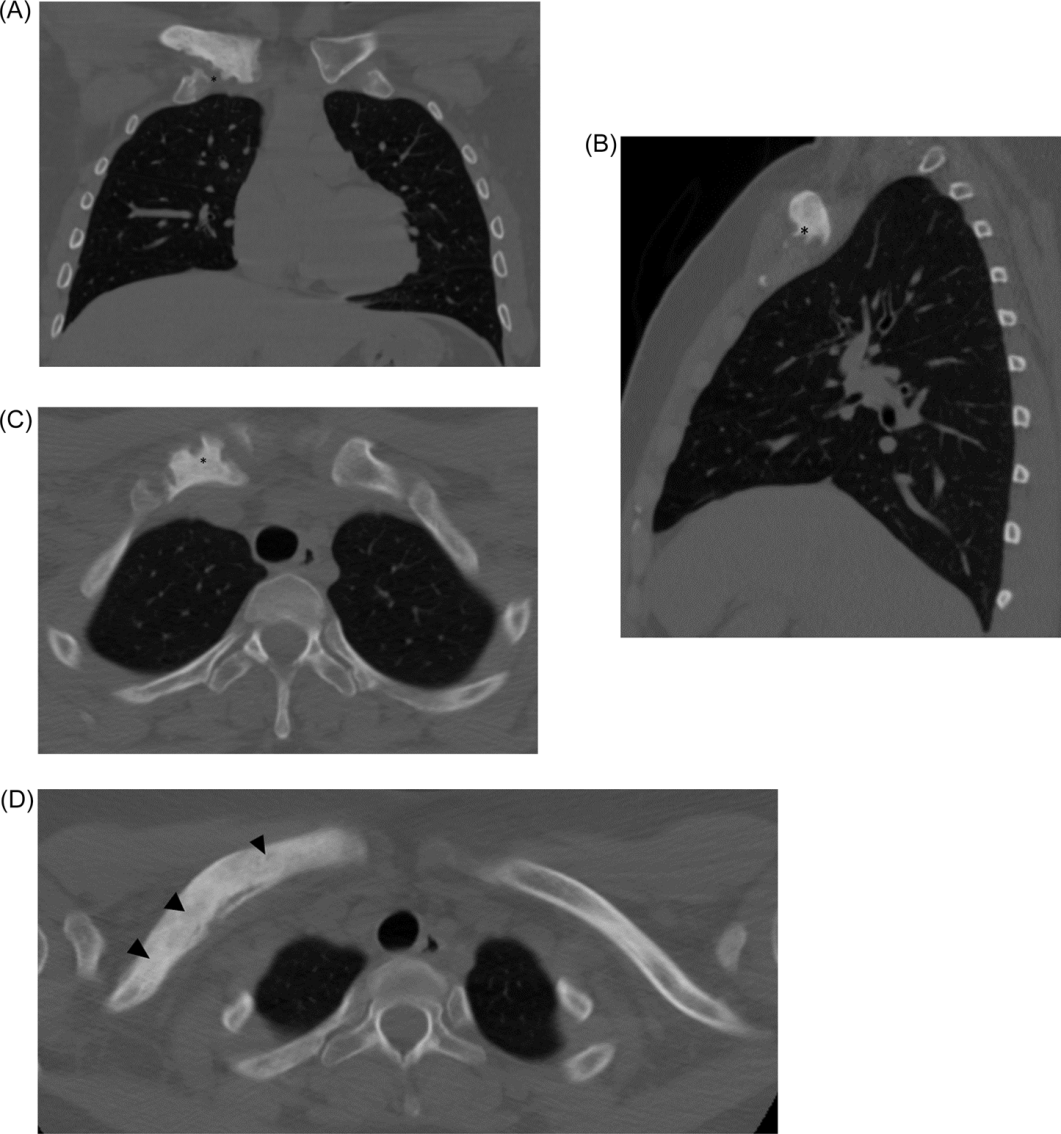
Chest CT without contrast obtained, September 2018, revealed a diffuse and expanded right clavicle with cortical breakthrough with scalloping and erosions. Coronal (A) and Sagittal (B) views depicting hook-like osteophytes in the inferior-medial clavicle (*). Axial (C, D) views demonstrating bony erosion (*) and marrow canal obliteration (black arrowheads).

A 29-year-old female was referred to our clinic in March 2021 for evaluation of right sided clavicle pain which had been affecting the patient for at least 3 years. As prior history, in 2018 the patient initially presented with medial right clavicle pain and palpable enlargement. The patient had no significant past medical or surgical history, and specifically denied any history of osteomyelitis, trauma to the right clavicle, or autoimmune diseases. At the time, she underwent a computed tomography (CT) of the chest without contrast, which demonstrated an area of increased bone sclerosis within an expanded right medial clavicle with evidence of cortical breakthrough as well as scalloping and erosions. Hook-like osteophytes were also noted. No associated soft tissue mass or pathological fracture were noted (Fig. 1). A myriad of diagnostic alternatives was considered at time, such as osteomyelitis, fibrous dysplasia and synovitis, acne, pustulosis, hyperostosis, osteitis (SAPHO) syndrome. Among the malignant possibilities, lymphoma, osteosarcoma and even metastatic disease were deemed possible, although the suspicious was low for the later give the patient's age. At this point, tissue sampling was recommended, and the patient underwent a right clavicle core needle biopsy, which demonstrated only lamellar trabecular bone and rendered nondiagnostic results despite an appropriate sample.

On initial evaluation in our clinic, in March 2021, the patient confirmed the pain in her right clavicle has continued intermittently since 2018. She had experienced episodes of increased pain approximately one time per month, usually occurring at nighttime or during her menses. These episodes were described as throbbing pain to the right clavicle associated with radiating pain to the right shoulder and neck. The patient stated that due to the severity of pain, she had to compensate with an antalgic position favoring the opposite side and now affecting her posture. On physical examination the patient had mild discomfort with palpation as well as bony irregularity noted to the right mid-clavicle. No lymphadenomegaly was present.

**Fig. 2:**
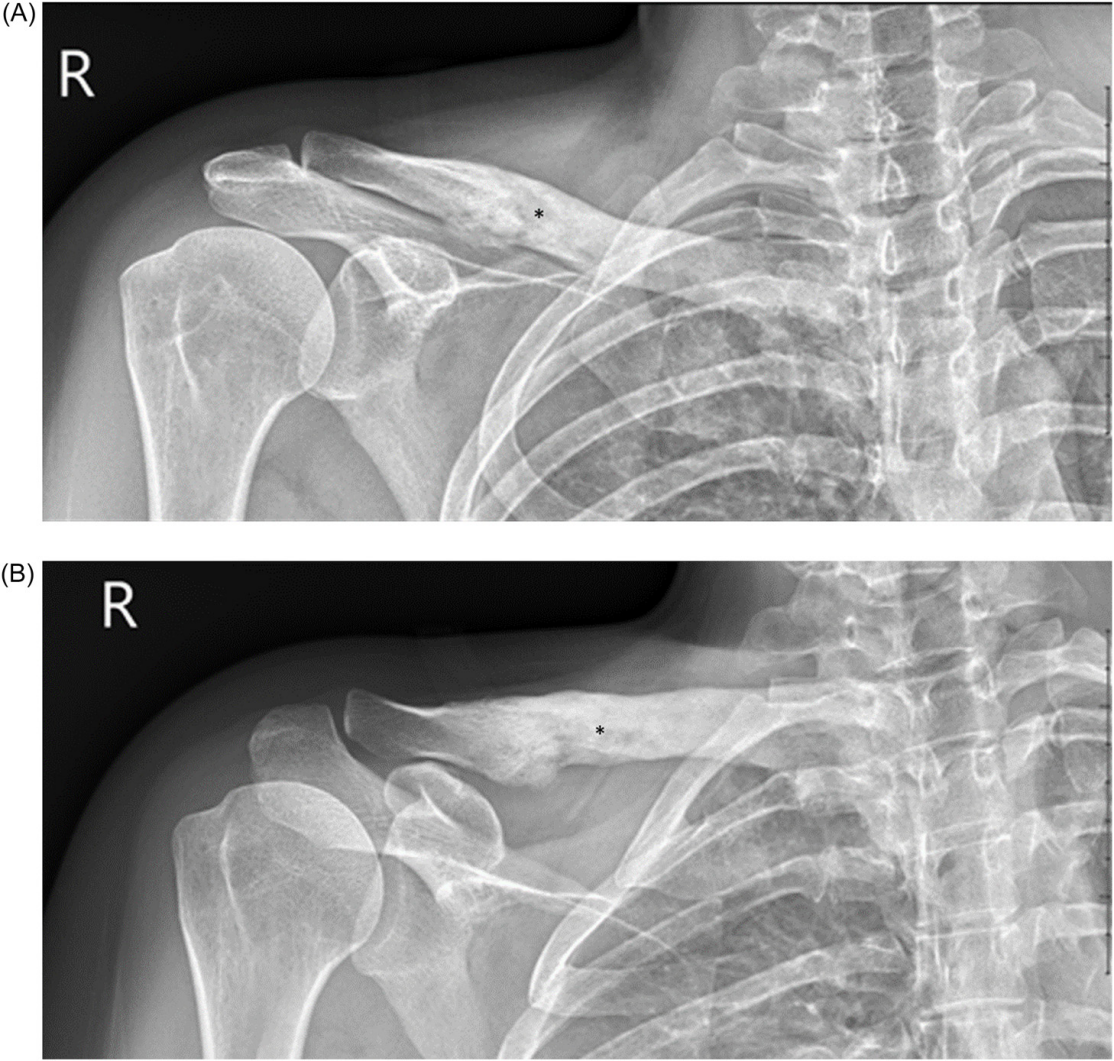
Radiographs of the right clavicle, AP (A) and AP with cephalad angulation (B) views revealing cortical irregularity, erosions and scalloping at the mid clavicle (*).

**Fig. 3:**
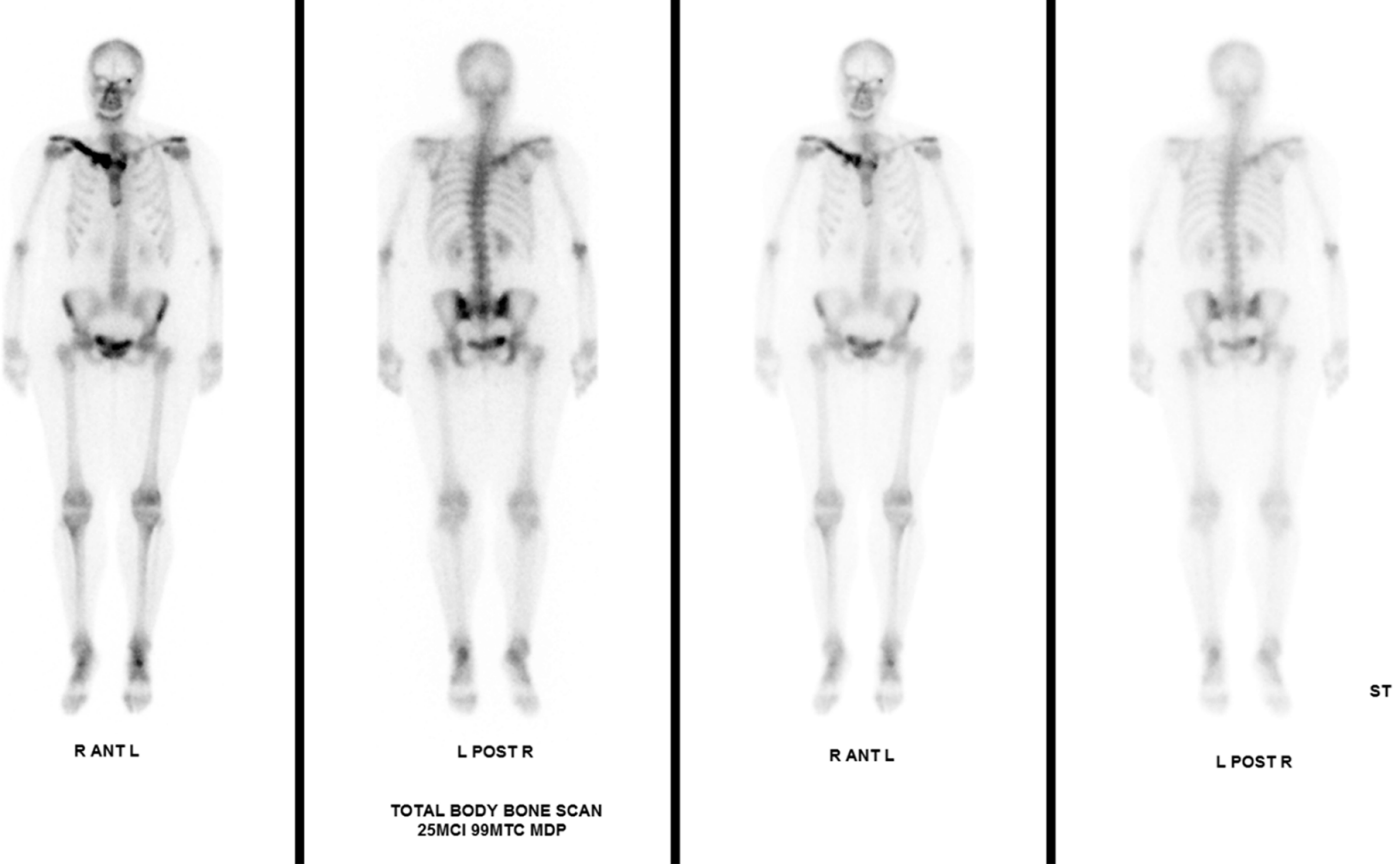
Nuclear medicine Tc 99 bone scan demonstrating intense increased uptake in the medial right clavicle. Physiological bone distribution is noted throughout the skeletal system otherwise.

Right clavicle radiographs obtained in March 2021 demonstrated evidence of cortical irregularity, erosions, and scalloping at the right mid-clavicle with interval worsening when compared to prior imaging from 2018 (Fig. 2). A whole-body bone scan was obtained then in April 2021 showing a solitary lesion in the right clavicle with significantly increased bony uptake corresponding to the area of sclerosis visualized on radiography (Fig. 3). Differential considerations at this time included the possibility of both benign or malignant neoplasms, as well as other non-neoplastic processes such as chronic recurrent multifocal osteomyelitis (CRMO), SAPHO syndrome, osteomyelitis, Paget's disease and fibrous dysplasia. As the previous needle biopsy in 2018 had returned a non-diagnostic specimen, at this time we recommended an open biopsy of the right medial clavicle.

The patient was taken to the operating room for open biopsy and a small transverse incision was made over the inferior aspect of the clavicle, centered over the observed lesion. At the time of the operation, harder sclerotic bone was noted to be fully obliterating the medullary canal. Multiple bony fragments were obtained, for both pathological as well as microbiological evaluation.

**Fig. 4:**
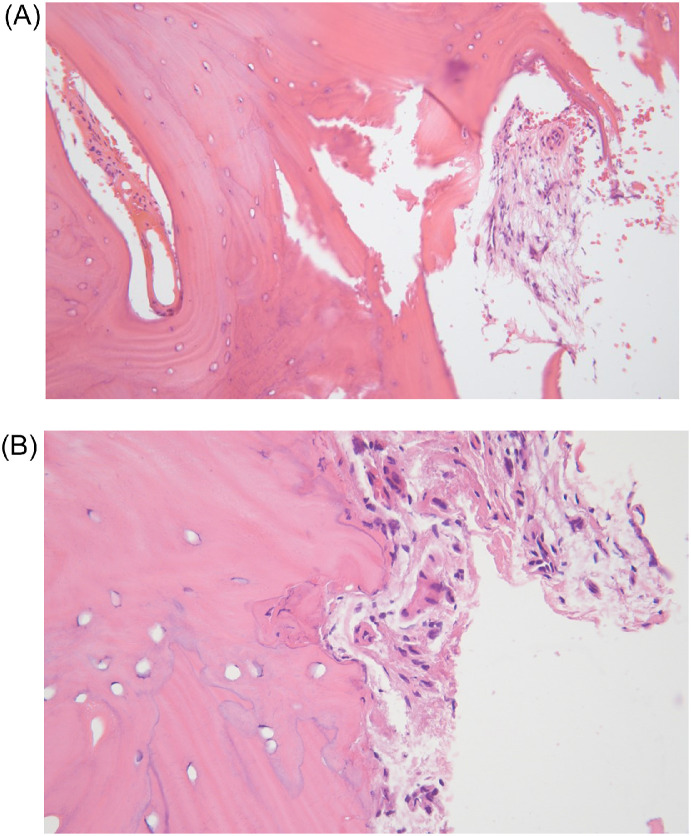
Histopathological examination of the right medical clavicle open bone biopsy. Low and high-power views (A: H&E, 200x; B: H&E, 400x) of benign appearing bone with marrow fibrosis and reactive woven and lamellar bone formation. No atypia is noted.

On gross examination the fragments obtained measured 1.1 × 1 × 0.2 cm and 3 × 1.5 × 0.3 cm, containing tan-pink to red irregular tissue and bone fragments. Pathological analysis of both specimens showed fragments of benign cortical and cancellous bone along evidence of prior remodeling with woven and lamellar reactive bone (Fig. 4). The bone marrow analysis demonstrated hemosiderin deposits and fibrosis as well as a chronic inflammatory infiltrate. The histopathological findings in this scenario and after clinical and radiographic correlation, favor the final diagnosis of condensing osteitis of the clavicle. Microbiological evaluation was unremarkable.

## Discussion

Condensing osteitis can be found in multiple bony locations such as the clavicle and the iliac bone. When diagnosed in the clavicle, it is mostly found on the medial third portion. This disease's imaging features often overlap with those of many different bone lesions such osteoid osteoma, osteoarthritis, metastases, osteosarcoma, Friedrich's disease, and bone islands, among others [11]. Even though the patient in the case debuted with the classical presentation for condensing osteitis, she remained undiagnosed for many years, even after undergoing an initial clavicle biopsy, which reveals the difficulties posed by this diagnosis.

On radiographic imaging studies Condensing Osteitis is observed as a homogeneously dense sclerotic lesion on the inferior/medial third of the clavicle present unilaterally. The sternoclavicular joint is usually spared and has normal appearance [11]. The presence of osteophytes can be noted occasionally and although those are often found in osteoarthritis, a common differential diagnosis in this scenario, there are no associated subchondral cysts, joint spaces narrowing, or erosions commonly seen in osteoarthritis patients [12]. On CT scans various degrees of bone marrow obliteration with soft tissue swelling surrounding the bone lesion are commonly observed as well as bone expansion and hook-like osteophytes over the medial end of the clavicle [12]. On Tc-99 radionuclide bone scans there is an increased uptake of the tracer in the sclerotic portions of the lesion; a finding that can induce a misdiagnose for Paget's disease [11,13]. Gallium and indium-labeled white blood cell scans are both normal appearance for this lesion [11]. MRI findings in T1-weighted sequences have revealed hypointense signal corresponding to the sclerotic portions of the lesions. T2-weighted sequences demonstrate the sclerotic portions of the lesion to be hypointense and isointense [5]. Prior case reports have found that T2-weighted sequences could additionally display regions of signal hyperintensity secondary to bone marrow edema [5]. Gadolinium contrast is not associated with enhancement of the sclerotic portion of the clavicle, while mild enhancement of the periosteum and surrounding soft tissue swelling can be present [5,13]. Although MRI can help determine the extent of bone marrow and soft tissue swelling associated with the Condensing Osteitis of the clavicle, it is not specific for the lesion and plain radiographs and CT scans are generally more useful to identify the lesion [14].

Histopathological features of the lesion demonstrate thickening of the trabeculae with lamellar and woven components as well as bone marrow obliteration with necrosis and fibrotic tissue [15]. These features are often present when there is evidence of bone remodeling with devitalization and necrosis of bone and periosteal reactions [1]. Unfortunately, these histopathological findings are often not specific enough for diagnosis and clinical and radiographic correlation is advised as well as a second opinion by a treating team with experience in rare bone conditions.

## Conclusion

Condensing Osteitis is a rare disease with a highly unspecific presentation and radiologic appearance, overlapping with a spectrum of other benign and malignant entities. A biopsy is necessary for diagnostic confirmation. Nevertheless, clinical and radiographic correlation are a requirement. A qualified multidisciplinary team with expertise in rare bone conditions becomes oftentimes essential to arrive at an accurate diagnosis.

## Patient Consent

Per the local Institutional Review Board consent was exempt due to this being the case of research involving the collection or study of existing data, documents, records, pathological specimens, or diagnostic specimen with the information being recorded by the investigator in such a manner that subjects cannot be identified, directly or through identifiers linked to the subjects. Nevertheless, the patient was informed and consented to publication.
